# Modelling the bearing and branching behaviors of 1-year-old shoots in apricot genotypes

**DOI:** 10.1371/journal.pone.0235347

**Published:** 2020-07-09

**Authors:** Martin Mészáros, Yann Guédon, Boris Krška, Evelyne Costes

**Affiliations:** 1 Research and Breeding Institute of Pomology Holovousy Ltd., Hořice, Czech Republic; 2 UMR AGAP, CIRAD, CIRAD-INRA-Montpellier SupAgro, Université de Montpellier, Montpellier, France; 3 Department of Fruit Growing, Faculty of Horticulture, Mendel University, Brno, Czech Republic; 4 UMR AGAP, INRA, CIRAD-INRA-Montpellier SupAgro, Université de Montpellier, Montpellier, France; IRHS, INRA, AGROCAMPUS-QUEST, Universite d’Angers, SFR 4207 QUASAV, FRANCE

## Abstract

In most temperate fruit trees, fruits are located on one-year old shoots. In *Prunus* species, flowers and fruits are born in axillary position along those shoots. The axillary bud fate and branching patterns are thus key components of the cultivar potential fruit production. The objective of this study was to analyze the branching and bearing behaviors of 1-year-old shoots of apricot cultivars and clones genetically closely related. Shoot structures were analyzed in terms of axillary bud fates using hidden semi-Markov chains and compared depending on the genotype, year and shoot length. The shoots were composed of three successive zones containing latent buds (basal zone), central flower buds (median zone) and vegetative buds (distal zone), respectively. The last two zones contained few associated flower buds. The zones length (in number of metamers) and occurrence strongly depended on shoot development in the two successive years. With decrease in the number of metamers per shoot, the last two zones become shorter or may not develop. While the number of metamers of the basal and distal zones and the number of associated flower buds correlated to the number of metamers of the shoot, the number of metamers of the median zone and the transition probability from the median to the distal zone were cultivar specific.

## Introduction

The architecture of temperate fruit trees is characterized by high-order branching structures composed of multiple axes of different lengths and complexities. This structure results from the differentiation and organogenetic activity of axillary meristems located at each metamer (composed of node its corresponding leaf and axillary bud plus the subtending internode) along the parent shoot. Axillary buds may have different fates leading to various shoot types with different rates and timings of outgrowth [1, 2, 3]. The relationship between the bud position and fate along the annual shoots determines the branching and flowering patterns [4, 5, 6]. Morphogenetic gradients during tree ontogeny are characterized by a decrease in shoot growth over successive years and branching orders, leading to a progressive simplification of branching pattern within trees [1, 7, 8, 9, 10]. Ontogeny can be tuned by the environmental stress factors, such as high temperature, water and nutrient availability [11, 12, 13, 14] affecting bud development [15, 16, 17]. Studying growth and branching patterns are thus crucial to understand tree bearing behaviors which are of agronomic importance [18, 19]. This knowledge has led to genotype classification [20, 21] and is also useful for breeding. Though the large majority of fruit are born on short (spurs) or medium (brindles) shoots, cultivar behaviors need to be further understood [22, 23, 24, 25].

Hidden semi-Markov chains (HSMCs) have been proposed to analyze branching and axillary flowering patterns over time and space [4, 5, 6, 10]. These models aim at segmenting the shoots into consecutive zones of homogenous composition in terms of axillary bud fates. HSMCs have been widely applied to compare different fruit species or cultivars, at different development stages, including apple [6, 10, 26], apricot [27] peach [28] and almond trees [14].

In Rosaceae fruit trees, branching and axillary flowering patterns largely vary among species [29]. While all these species are characterized by rhythmic growth [1], they differ in the location of reproductive and vegetative buds and therefore in the shoot structure [29]. While flowers are in terminal position in apple tree [30], *Prunus* species are characterized by axillary flower bud position along annual shoots [28, 31]. Flowers are located in upper position along the shoot in apricot, peach and almond trees, whereas they are located at the basis position of the shoot in cherry trees [29]. Moreover, the majority of *Prunus* species is able to produce additional flowers associated to either vegetative or central flower buds. These differences in axillary bud fates lead to different branching patterns in peach [28], apricot and cherry trees [32]. In apricot tree, a sympodial branching (i.e. branching from an axillary bud, after the mortality of the terminal meristem; for more detail description of the terminology, see also [1, 33]), has been related to a frequent terminal bud mortality [34]. While differences in branching pattern can be found among cultivars of a species [6, 10, 31], there is no evidence so far of a possible variability among clones of a same cultivar.

In the present study, the axillary bud fates were analyzed along annual shoots on a set of apricot cultivars, including the standard apricot cultivar ‘Velkopavlovická’, that is the most cultivated in Czech Republic and a set of its clones [35] using HSMCs. The differences in the shoot structures were analyzed with respect to three effects: genotype, shoot length and successive years. The objective of the study was to deepen our understanding of branching and flowering patterns in adult apricot trees by the detail description of vegetative and reproductive bud development along annual shoots in a set of cultivars and clones.

## Materials and methods

### Plant material and orchard environment

The experimental orchard was planted in spring 2001 at 6 x 3.5 m spacing at the Horticultural faculty of Mendel University in Lednice (Czech Republic). The orchard was situated in clay-loam black soils. All trees were grafted on apricot seedlings from cv Yulskiy (with middle Asian origin). After planting the trees were pruned in open vase. However, during the trial the trees were not pruned and the orchard was left without irrigation.

Eight genotypes of apricot trees (*Prunus armeniaca* L.) with similar growth and fruiting habits were considered for this study. Among them, we considered four cultivars (cvs): ‘Bergeron’, LE-97, NS-2 and ‘Velkopavlovická’ (VP), represented by reference clone VP-LE-12/2 [35]. This reference clone was compared with four other VP clones (Doc. Blatny, LE-111, LE-130 and LE-285). They all belong to Group 5, which corresponds to relatively small trees which axes bend easily and bear fruit mainly on medium shoots [21]. They are characterized by equilibrium among different shoot types that all bear fruit and are considered as the most suitable for agronomic cultivation. ‘Bergeron’ is a well-known random seedling from Rhône valley, France. Genotype LE-97 was obtained by random pollination of VP cv and NS-2 originates from Novi Sad, Serbia as a nature population from Vojvodina region. ‘Velkopavlovická’ is an apricot originating from South Moravia (Czech Republic) near Velké Pavlovice. In previous studies, ‘Velkopavlovická’ was considered as a clone of the cultivar ‘Magyar Kajszi’ belonging to the group of ‘Hungarian Best’ cultivars [36, 37].

In the two years during which the observed shoots had developed (2010 and 2011), the maximum, minimum and mean temperatures were 21.4 °C, −3.1 °C, 9.4 °C and 24.3 °C, −0.9 °C, 10.6 °C, respectively. The dynamics over each year was typical of temperate climate (S1 Fig). The cumulative rainfalls during these two years were 710.4 mm and 442.4 mm, respectively with a maximum in May 2010 and in July for 2011.

### Shoot observations

Four to six trees per genotype were observed in two consecutive years, 2011 and 2012. Six lateral branches (one or two per tree) per genotype were selected (S2 Fig) according to the following rules: (1) the branches were situated on periphery of the trees at 1.5–2.0 m from the ground, (2) the length of three-year-old part of these branches were in the range of 150–300 mm, (3) their basal angle did not exceed -10 to 45° with the horizontal plane, (4) these branches were at the third branching order at the tree level and (5) the shoot in the continuation of each branch was longer than 10 cm. Along these branches, all annual shoots developed over three years (from three- to one-year-old shoots) were described retrospectively at the metamer scale twice, i.e. in 2011 and 2012 (S3 Fig). Moreover, the year during which each shoot had developed was noted to enable studying the year effect.

Bud fates were described node by node along the shoots. The central buds were coded “L”, “V” or “F” for latent (i.e. buds that do not outgrow in the next year), vegetative (i.e. buds that are more developed than latent buds and are expected to outgrow a vegetative shoots in the next year) or floral fate, respectively and the number of associated flower buds noted (0, 1 or ≥ 2 associated flower buds). During the observation years, the development of flowers into fruit was assessed as the mean number of fruit per shoot. However, because this number was low, this variable was not considered afterwards in the modelling approach.

### Data analysis and model building

The central bud fate and the number of associated flower buds were organized as bivariate sequences indexed by node rank. Following previous studies [5, 6, 26], a HSMC was built for each genotype on the basis of bivariate sequences pooled for the different orders and growing years considering all shoot together. A HSMC is a two-scale segmentation model whose aim is to segment observed sequences into successive homogeneous zones both in terms of central bud fate and number of associated flower buds. In this framework, the succession and length (in number of metamers) of zones (coarse scale not directly observable) are represented by a non-observable semi-Markov chain, whereas the central bud fate and the number of associated flower buds within each zone (fine scale) are represented by categorical distributions attached to each state of the semi-Markov chain. A semi-Markov chain is defined by three subsets of parameters:

1. Initial probabilities to model which is the first zone in the shoot,
2. Transition probabilities to model the succession of zones along the shoot,
3. Occupancy distributions attached to non-absorbing states (a state is said to be absorbing if, after entering this state, it is impossible to leave it) to model lengths of zones in number of nodes/*metamers*.

A HSMC adds a fourth subset of parameters to the three subsets of parameters previously defined for the underlying semi-Markov chain:

1. Categorical observation distributions to model either the central bud fate or the number of associated flower bud. This entails that a probability mass was directly estimated for each possible category of each observed variable.

A ‘left-right’ HSMC composed of successive transient states followed by a final absorbing state was built on the basis of bivariate sequences corresponding to a given genotype. A state is said to be transient if after leaving this state, it is impossible to return to it. In a ‘left-right’ model, the states are thus ordered and each state can be visited at most once. In this study, it was chosen to assume that the end of an observed sequence systematically coincides with the transition from the current state to an extra absorbing ‘end’ state modeling implicitly the growth cessation (no observation model is associated with this end state; see [10]). Hence, at the end of an observed sequence, the process systematically jumps to the absorbing end state. In this way, it was possible to model explicitly the length of the most distal zone. Each estimated model was used to compute the most probable state sequence for each observed sequence [38]. This restored state sequence can be viewed as the optimal segmentation of the corresponding observed sequence into sub-sequences, each corresponding to a given zone. On the basis of the optimal segmentations of the observed sequences, “contextual” model parameters (e.g. probability of transition between two zones or distribution of the central bud fate for a given zone) or characteristic distributions (e.g. probability of occurrence of a given zone) specific to 2010 or 2011 shoots were extracted [10] in order to compare them.

### Comparison of shoots depending on the genotype, year and shoot types

To compare the shoot branching structures among genotypes and years, the shoots were classified into three types depending on their length: a) short shoots (SS) up to 2 cm, b) medium shoots (MS) from 2.1–10 cm and c) long shoot (LS) above 10 cm. Such classification was based on previous studies which considered axes polymorphisms based on the concepts of preformation and neoformation [33]. Even though overlap between classes remained (S1 Table), this approach allowed us to compare the “contextual” model outputs with respect of a given class of shoot length. For each shoot type, the year and genotype effects on the number of metamers per shoot and per zone, the number of associated flower buds and fruit per shoot were analyzed using the Kruskal-Wallis test (ANOVA by ranks), and further compared among genotypes, years or shoot types using the Wilcoxon-Mann-Whitney test. The probabilities of zone occurrence were compared using a Pearson χ^2^ test. After the optimal segmentation of all the observed sequences in successive zones, the relationship between the number of metamers of two zones as well as between the number of metamers of each zone and the total number of metamers of the shoot was analyzed using Spearman rank correlation coefficients. Among all genotypes, the longevity of the lateral shoots along the branches was analyzed depending on the year of shoot development using the Pearson χ^2^ test.

The HSMC and all associated statistical methods are implemented in the Structure Analysis module of the Openalea software platform [39].

## Results

### Number and types of shoots

**Fig 1:**
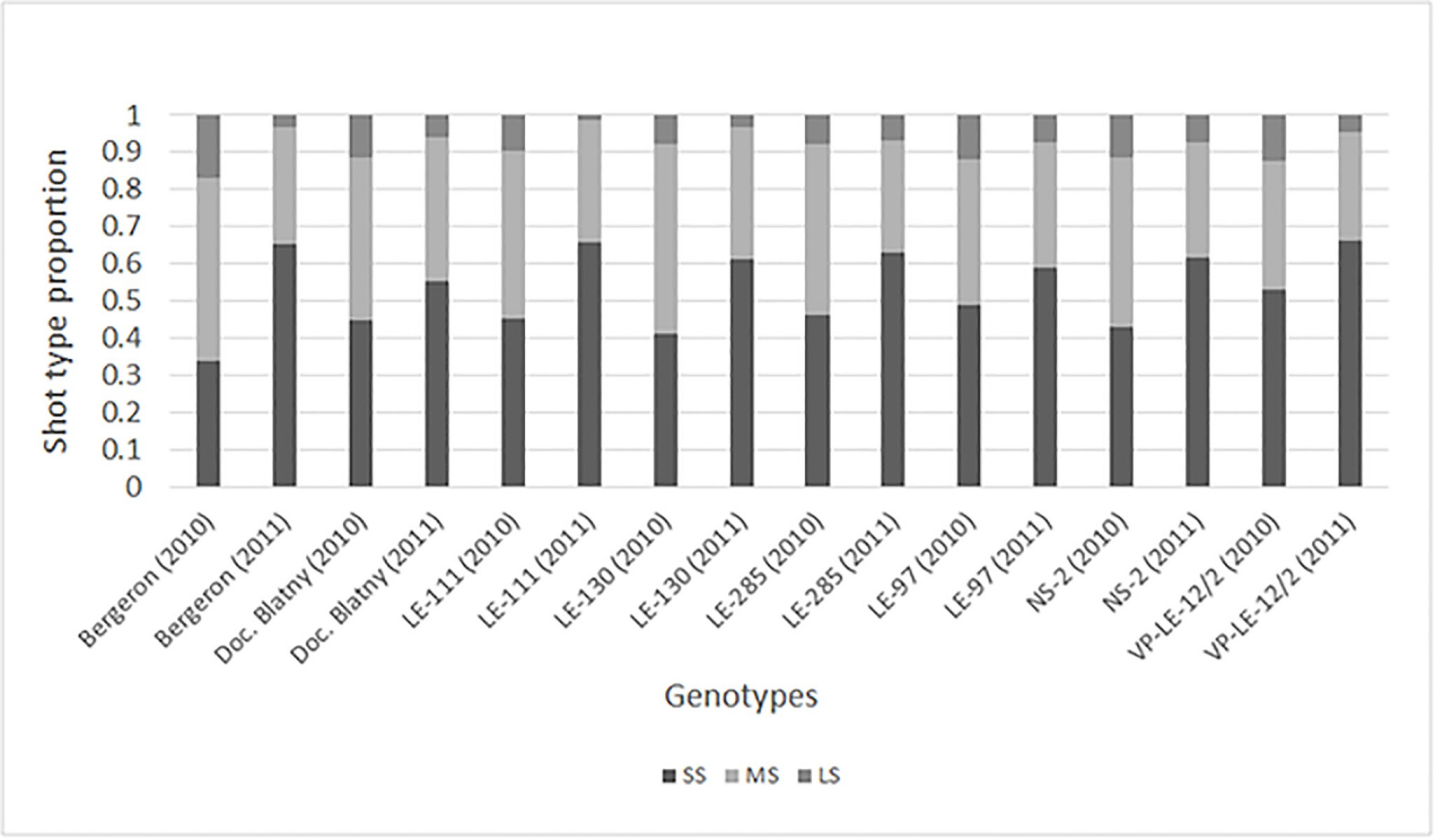
Proportion of shoot types composing branches of cultivars ‘Bergeron’, NS-2, ‘Velkopavlovická’ (VP-LE-12/2), LE-97 and VP clones Doc. Blatny, LE-111, LE-130 and LE-285. Branch development were observed in two successive years (2010 and 2011); short shoots (SS), medium shoots (MS), long shoots (LS).

The total number of developed shoots on the selected branches ranged from 81 to 153 and 129 to 227 depending on the genotype, in 2010 and 2011, respectively. Considering the relatively old tree age, short and medium shoots were in higher proportions than long shoots. In 2010, the proportion of shoot types ranged from 0.34 to 0.53 of SS, from 0.35 to 0.51 of MS and from 0.08 to 0.17 of LS depending on the genotype (Fig 1), with ‘Bergeron’ exhibiting the highest proportion of MS and LS. In 2011, all genotypes developed a similar proportion of shoot types, with a majority of SS, some MS and few LS, even though ‘Bergeron’ had the highest increase of SS proportion and LE-97 the lowest. This means that the proportion of MS and LS significantly decreased between 2010 and 2011 whereas SS significantly increased (according to a Pearson’s χ^2^ test, p = 8.51E^-20^).

### Estimation of the hidden semi-Markov chains

**Fig 2:**
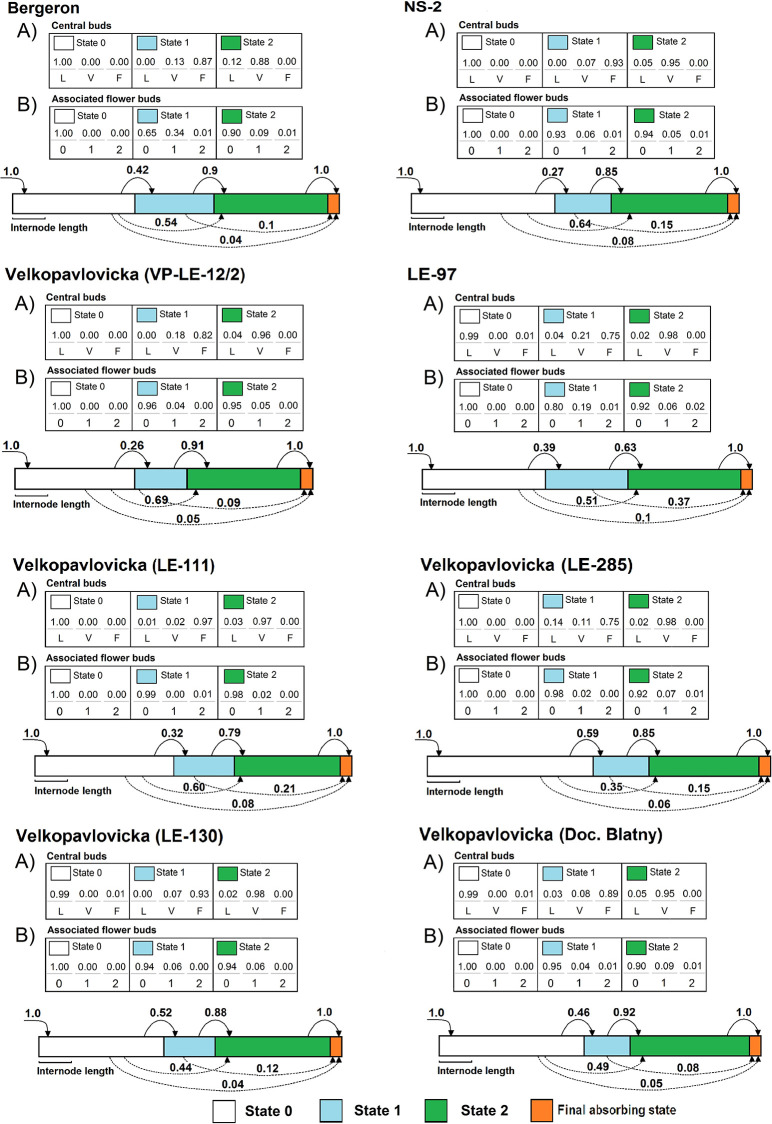
Schematic representation and parameter values of the hidden semi-Markov chains estimated on one-year-old shoots of cultivars ‘Bergeron’, NS-2, ‘Velkopavlovická’ (VP-LE-12/2), LE-97 and VP clones Doc. Blatny, LE-111, LE-130 and LE-285 developed in 2010 and 2011. Top part (tables): Observation distributions and proportions of (A) the central bud fate (L—latent bud, V—vegetative bud, F—flower bud) and (B) number of associated flower buds (0–no lateral flower bud, 1–one associated flower bud, 2–two or more associated flower buds) in each state; Bottom part (diagrams): states are represented by colored rectangles whose length is proportional to the mean number of metamers of the state, when present; initial probabilities are represented by arrows entering in the first state, transition probabilities are represented by arrows between states, with the transition probability indicated nearby.

The shoots varied from 2 to 26 metamers, depending on the type, genotype and year (S1 Table). The HSMC estimated for each genotype were composed of three transient states modeling three successive zones and a final absorbing end state (see Fig 2). The first (basal) zone was composed of latent buds, exclusively. The second (median) zone contained mainly central flower buds with some associated flower buds. The third (distal) zone contained mainly vegetative shoots sometimes with few associated flower buds. The mean number of metamers was similar for the basal and distal zones, these zones having a higher mean number of metamers than the median zone. The first zone containing latent buds was always present whereas the two other zones may be skipped leading to shoots without flowering zone and/or shoots without distal vegetative zone depending on the genotype.

### Comparison of shoot structure among genotypes

The genotypes differed in the mean number of metamers of the basal and median zones exhibiting highly significant differences (Table 1). Among cvs, the highest mean number of metamers in the basal zone, over the two years, was observed in NS-2. Among the VP clones, the variability was relatively large with the highest mean value found in LE-285 and the lowest in VP-LE-12/2. Despite a relatively low variability in the number of metamers of this zone (less than 2), the groups overlapped between cvs and clones (Table 1). For the median zone, the highest mean number of metamers over the two years was observed in ‘Bergeron’ and LE-97 for the cvs, whereas the lowest was in Doc. Blatny among clones, even though similar to all the VP clones.

**Table 1:** Mean number of metamers in the basal and median zones (when present), mean number of metamers per shoot and mean number of associated flowers and fruit per shoot depending on the genotype (cvs and VP clones) and year of shoot development. Genotype and year effects were analyzed using a Kruskal-Wallis test. Comparisons among genotypes and years were performed with a Wilcoxon-Mann-Whitney test. Different letters in a same variable indicate significant difference (p < 0.05) among cvs and VP clones and between years. The distributions extracted for the two years jointly were compared among genotypes separately.

	Genotypes	Basal zone			Median zone			Shoot		Associated flower buds		Fruit	
		2010	2011	2010–2011	2010	2011	2010–2011	2010	2011	2010	2011	2010	2011
**Cultivars**	**Bergeron**	4.22 bcde	3.57 fg	3.83 c	2.79 a	1.64 bc	2.50 a	9.75 ab	7.26 c	1.27 a	0.44 bc	0.96 a	0.01 e
	**LE-97**	3.97 cdef	3.68 efg	3.80 c	2.84 a	1.39 cd	2.57 a	9.01 ab	7.37 c	1.32 a	0.01 d	0.63 b	0.02 e
	**NS-2**	3.56 fg	4.94 b	4.38 b	1.88 b	-	1.88 b	8.77 b	8.53 b	0.64 b	0.02 d	0.50 bcd	0.00 e
**VP reference clone**	**VP-LE-12/2**	3.23 h	3.92 def	3.68 c	1.83 bc	1.20 d	1.66 bc	7.84 bc	7.34 c	0.47 bc	0.03 d	0.58 bc	0.00 e
	**Doc. Blatny**	3.88 defg	4.75 bc	4.41 b	1.77 bc	1.27 d	1.56 c	8.83 b	8.72 b	0.55 bc	0.31 bc	0.59 bc	0.01 e
	**LE-111**	4.41 bcd	4.09 cdef	4.22 bc	1.87 b	1.00 d	1.86 bc	9.01 ab	7.29 c	0.16 c	0.00 e	0.53 bcd	0.00 e
	**LE-130**	4.55 bcd	3.42 gh	3.88 c	1.56 bcd	1.71 bc	1.64 bc	8.59 b	7.29 c	0.20 c	0.29 bc	0.22 d	0.00 e
	**LE-285**	5.96 a	4.67 bcd	5.13 a	1.86 bc	1.60 bc	1.71 bc	10.29 a	8.78 b	0.22 c	0.22 c	0.30 cd	0.02 e
	**Genotypic effect**	***			***			ns.		***		***	
	**Year effect**	*			***			*		***		***	

The statistical difference among genotypes and years (particular variable) was signed with different letters (p = 0.05) with the values.

ns. = not significant,

* = significance at α = 0.05,

** = significance at α = 0.01,

*** = significance at α = 0.001.

Differences among genotypes were also found in the transition probabilities between the median and distal zones (Fig 2), and consequently in the occurrence of these zones (Table 2). The median zone occurred more frequently in cvs ‘Bergeron’ and LE-97, while it occurred less frequently in NS-2 and VP-LE-12/2 over the two years. Among clones of ‘Velkopavlovická’, the occurrence of the median zone was more frequent in LE-130 and LE-285 than in LE-111 and VP-LE-12/2. Almost all cultivars were characterized by a relatively high frequency of occurrence of the distal zone except LE-97. The most frequent occurrences of the distal zone among VP clones were found in VP-LE-12/2 and Doc. Blatny, whereas the less frequent occurrences were in LE-111 and LE-285.

**Table 2:** Frequency of occurrence of the median and distal zones per shoot depending on the genotype and year of shoot development. The year effect was tested by Pearson χ2 test. Comparisons among genotypes (cvs and VP clones) and between years were performed with a Pearson χ2 test. Different letters in a same variable indicate significant difference (p < 0.05). The two-year proportion of zone occurrence among cultivars and VP clones was compared separately.

	Genotype	Median zone			Distal zone		
		2010	2011	2010–2011	2010	2011	2010–2011
**Cultivars**	**Bergeron**	0.76 a	0.17 f	0.41 b	0.92 ab	0.91 ab	0.91 ab
	**LE-97**	0.74 ab	0.11 fg	0.36 bc	0.66 d	0.86 bc	0.78 d
	**NS-2**	0.66 abc	0.00 h	0.27 de	0.85 abc	0.89 abc	0.87 abc
**Velkoplavlovicka clones**	**VP-LE-12/2**	0.51 d	0.10 g	0.24 e	0.89 abc	0.94 a	0.92 a
	**Doc. Blatny**	0.62 bc	0.30 e	0.42 b	0.90 abc	0.91 ab	0.91 ab
	**LE-111**	0.75 ab	0.01 h	0.32 cd	0.83 c	0.87 abc	0.85 c
	**LE-130**	0.52 d	0.49 d	0.50 ab	0.87 abc	0.92 ab	0.90 abc
	**LE-285**	0.58 cd	0.48 d	0.51 a	0.83 c	0.88 abc	0.86 bc
	**Year effect**	***		-	***		-

The statistical difference among genotypes and years (particular variable) was signed with different letters (p = 0.05) with the values.

ns. = not significant,

* = significance at α = 0.05,

** = significance at α = 0.01,

*** = significance at α = 0.001.

The proportions of central flower buds within the median zone differed depending on the genotype (Fig 2), the highest being observed in NS-2 whereas the lowest were in LE-97. The metamers with associated flower buds were few and scattered between the median and distal zones in all genotypes. However, the number of metamers with associated flower buds was higher in the median zone than in the distal one (Fig 2), especially in cvs ‘Bergeron’ and LE-97. In all the genotypes, metamers with a single associated flower bud were more numerous than those with several associated flower buds.

### Comparison between the two observed years

By comparing shoots developed in the different years, further differences were found. The mean number of metamers per shoot decreased significantly between the two years in cvs ‘Bergeron’ and LE-97, as well as in VP clones LE-111, LE-130 and LE-285 (Table 1). In contrast, it remained similar between years in NS-2, VP clones Doc. Blatny and VP-LE-12/2. These changes impacted the shoot structure, especially, the mean number of metamers per zone. The length of the basal zone decreased with the years in cvs and clones with a decreasing number of metamers per shoot, whereas it was higher in those which shoot length remained stable. A decrease in distal zone length was also slightly significant between the two years, but only when all genotypes were considered together (p value = 0.04). The mean number of metamers in the median zone as well as the mean number of associated flower buds and fruit significantly decreased in a majority of genotypes between the two years, except in VP clones LE-130 and LE-285 (Table 1), having similar number of flower buds and fruit per shoot in both years.

The changes in shoot length between years also impacted the occurrence of the median and distal zones. The frequency of occurrence of the median zone varied between years depending on cvs and VP clones and significantly decreased between the two years in a majority of genotypes (Table 2). The distal zone occurred less frequently in cv LE-97 whatever the year, and in VP clones LE-111 and LE-285 when the two years were considered jointly (Table 2). The occurrence of the distal zone changed only slightly between the two years (Table 2), with significant increase between the years in LE-97 only.

### Comparison of the shoot organization among different shoot types

The mean number of metamers per shoot mirrors the shoot category, increasing from SS to MS and from MS to LS. It did not change between the two years in SS and MS, but tend to decrease in LS (S1 Table). There are few significant correlations between the numbers of metamers of each zone. Significant correlations were found negative between the basal and the median zones and the median and the distal zones while positive between the basal and the distal zones (Table 3). It should be noted that these significant correlations coefficients are often in absolute value just slightly above the limit corresponding to no correlation. The total number of metamers of the shoot correlated with those in the basal and the distal zones but not the median zone (Table 3), except in the two cultivars Bergeron and LE-97 which exhibited the highest mean number of metamers per median zone.

**Table 3:** Spearman rank correlation coefficients between the number of metamers of pairs of zones chosen among basal, median and distal zone, and the number of metamers of each zone and the total number of metamers of the shoot, depending on the genotypes (cvs and VP clones) and years.

	Genotype	Year	Basal vs. Median	Median vs. Distal	Basal vs. Distal	Basal vs. Shoot	Median vs. Shoot	Distal vs. Shoot	Limits
**Cultivars**	Bergeron	2010	ns.	ns.	0.3	0.57	0.36	0.8	±0.16
	Bergeron	2011	ns.	ns.	0.18	0.55	0.19	0.83	±0.13
	LE-97	2010	ns.	ns.	ns.	0.26	0.38	0.73	±0.17
	LE-97	2011	−0.15	ns.	ns.	0.3	ns.	0.87	±0.14
	NS-2	2010	ns.	−0.2	0.28	0.5	ns.	0.9	±0.17
	NS-2	2011	-	-	0.39	0.8	-	0.84	±0.14
**Velkoplavlovicka clones**	VP-LE-12/2	2010	ns.	ns.	ns.	0.49	ns.	0.85	±0.22
	VP-LE-12/2	2011	ns.	ns.	0.21	0.7	ns.	0.82	±0.16
	Doc. Blatny	2010	−0.29	ns.	ns.	0.47	ns.	0.82	±0.19
	Doc. Blatny	2011	ns.	ns.	0.22	0.76	0.2	0.75	±0.15
	LE-111	2010	−0.53	−0.26	ns.	0.53	−0.22	0.86	±0.2
	LE-111	2011	ns.	ns.	ns.	0.65	ns.	0.64	±0.17
	LE-130	2010	−0.28	ns.	0.3	0.71	ns.	0.81	±0.16
	LE-130	2011	−0.21	−0.3	ns.	0.44	ns.	0.78	±0.14
	LE-285	2010	ns.	ns.	ns.	0.75	ns.	0.68	±0.19
	LE-285	2011	−0.19	ns.	ns.	0.62	ns.	0.64	±0.14

ns. = correlation coefficients not significantly different from 0 at α = 0.05 which are between the two limits indicated in the last column.

The median zone was not present for cv NS-2 in 2011.

The shoot types impacted the mean number of metamers per zone. The basal and median zone tended to be lower in SS than in MS and LS (Table 4). However, the median zone tended to have a slightly higher number of metamers in MS than in LS, at least in Doc. Blatny and LE-130 (Table 4). Consistently with the strong relationship found between the number of metamers per shoot and the number of metamers of the distal zone, the mean number of metamers in that zone differs significantly among shoot types. The correlation between the total number of metamers per shoot and the number of associated flower buds per shoot is moderate with *r* = 0.37, confirming the increase of the number of associated flower buds per shoot with the shoot length, with differences among cvs and clones (Table 4). The highest mean number of associated flower buds in all shoot types was in ‘Bergeron’ whereas, the mean number of associated flowers was low in all the VP clones, especially on SS. The mean number of fruit was usually lower in SS and the genotypes differed in MS and LS only (Table 4).

**Table 4:** Mean number of metamers of the basal, median and distal zones (when present), and mean number of associated flower buds and fruit per shoot type depending on the genotypes (cvs and VP clones) on shoots developed in years 2010 and 2011. Shoot type effect was estimated with a Wilcoxon-Mann-Whitney test. Comparisons among genotypes and shoot types for a given zone were performed using a Wilcoxon-Mann-Whitney test. Different letters for a given zone indicate significant difference (p < 0.05).

	Genotype	Basal zone			Median zone			Distal zone			Associated flower buds			Fruit		
		SS	MS	LS	SS	MS	LS	SS	MS	LS	SS	MS	LS	SS	MS	LS
**Cultivars**	**Bergeron**	3.32 d	4.25 cd	5.09 abc	1.76 c	2.87 ab	2.78 ab	1.99 e	3.88 d	9.94 b	0.06 f	0.89 b	4.55 a	0.15 cde	0.63 ab	0.82 a
	**LE-97**	3.56 cd	4.11 cd	4.00 cd	1.79 c	3.38 a	3.20 a	1.37 e	4.26 cd	11.23 ab	0.01 g	0.65 c	3.29 a	0.15 cde	0.41 ab	0.35 ab
	**NS-2**	3.72 cd	4.98 bc	5.86 ab	1.76 c	1.93 c	2.17 bc	1.63 e	4.74 cd	12.48 a	0.01 g	0.35 d	1.59 b	0.14 cde	0.32 abc	0.14 cde
**VP reference clones**	**VP-LE-12/2**	3.25 d	4.34 cd	4.59 bcd	1.50 c	1.95 c	1.40 cd	1.95 e	4.97 c	9.59 b	0.02 g	0.18 e	1.53 b	0.10 de	0.30 a-d	0.65 ab
	**Doc. Blatny**	3.96 cd	4.87 bc	4.95 bc	1.46 cd	1.70 c	1.27 d	1.65 e	4.46 cd	12.64 a	0.01 g	0.27 e	3.59 a	0.12 de	0.33 ab	0.50 ab
	**LE-111**	3.66 cd	5.05 bc	4.45 bcd	2.00 bc	1.75 c	1.50 c	1.73 e	4.35 cd	11.36 ab	0.02 g	0.06 f	0.73 bc	0.22 b-e	0.25 a-d	0.09 de
	**LE-130**	3.38 d	4.37 cd	5.17 abc	1.70 c	1.65 c	1.00 d	1.56 e	4.37 cd	9.00 b	0.02 g	0.35 d	1.89 b	0.10 de	0.08 de	0.11 de
	**LE-285**	4.56 bcd	5.74 ab	6.67 a	1.69 c	1.75 c	1.56 c	1.53 e	4.18 cd	12.90 a	0.01 g	0.13 e	3.19 a	0.08 e	0.21 b-e	0.05 e
	**Shoot type effect**	***			**			***			***			***		

The statistical difference among genotypes and shoot types (particular variable) was signed with different letters (p = 0.05) with the values.

ns. = not significant,

* = significance at α = 0.05,

** = significance at α = 0.01,

*** = significance at α = 0.001.

SS—short shoots, MS—medium shoots, LS—long shoots.

The shoot types also impacted the frequency of occurrence of the zones. The frequency of shoots with median and distal zone tended to be lower in SS than in MS and LS, but with differences depending on the genotype (Table 5).

**Table 5:** Frequency of occurrence of the median and distal zones depending on the shoot type and genotypes (cvs and VP clones) on shoots developed in years 2010 and 2011. The difference among genotypes and shoot types were estimated for each variable with a Pearson χ2 test.

	**Median zone**				
	**Genotype**	SS	MS	LS	Shoot type effect (SS/MS/LS)
**Cultivars**	**Bergeron**	0.245 cd^1^	0.565 ab	0.697 a	y/x/x^2^
	**LE-97**	0.328 c	0.419 bc	0.323 bc	x/x/x
	**NS-2**	0.216 d	0.364 c	0.207 c	y/x/x
**Velkoplavlovicka clones**	**VP-LE-12/2**	0.204 d	0.284 c	0.294 bc	x/x/x
	**Doc. Blatny**	0.336 bc	0.519 b	0.500 ab	y/x/xy
	**LE-111**	0.281 cd	0.376 c	0.364 bc	x/x/x
	**LE-130**	0.479 a	0.537 ab	0.444 ab	x/x/x
	**LE-285**	0.436 ab	0.657 a	0.429 ab	y/x/y
	**Distal zone**				
	**Genotype**	SS	MS	LS	Shoot type effect (SS/MS/LS)
**Cultivars**	**Bergeron**	0.840 ab	0.993 a	1 a	y/x/x
	**LE-97**	0.635 c	0.960 b	1 a	y/x/x
	**NS-2**	0.767 b	0.992 a	1 a	y/x/x
**Velkoplavlovicka clones**	**VP-LE-12/2**	0.871 a	1.000 a	1 a	y/x/x
	**Doc. Blatny**	0.832 ab	0.991 ab	1 a	y/x/x
	**LE-111**	0.773 b	0.953 b	1 a	y/x/x
	**LE-130**	0.819 ab	0.993 a	1 a	y/x/x
	**LE-285**	0.761 b	0.990 ab	1 a	y/x/x

^1^The statistical difference among genotypes (columns) is indicated by different letters (p = 0.05).

^2^Statistical difference among shoot types in each genotype (rows) is indicated by letters as well (x, y, z).

SS—short shoots, MS—medium shoots, LS—long shoots.

### Shoot life span

In both years (2010 and 2011), the shoots longevity along the observed branches depended on their length. LS were always alive two years after their development whereas a proportion between 0.80 and 0.95 of MS was alive one year after their development, whatever the genotypes and years. In contrast, the proportion of SS remaining alive one year after their development was much lower than in MS and LS. SS developed in 2009 remained alive in 2010 with a proportion ranging from 0.64 (NS-2) to 0.23 (LE-97; Table 6). No significant difference was observed in VP clones for this trait. SS developed in 2010 remained alive in 2011 with a proportion ranging from 0.79 (VP-LE-12/2) to 0.22 to (LE-97). The reference clone VP-LE-12/2 had a higher proportion of living SS than all other VP clones. The proportion of shoots remained alive over two years varied from 0.57 (VP-LE-12/2) to 0.17 (LE-97). Here again the ranking of genotypes with respect to SS survival was similar with VP-LE-12/2 having the highest survival and LE-97 the lowest.

**Table 6:** Proportion of short shoots developed in 2009 and 2010 still alive along the branch axis in 2010 (2009/2010) and 2011 (2010/2011), respectively. The survival of all shoots, either one and two years old, developed either in 2009 or 2010, and observed in 2011 are represented with (2009-10/2011). Comparisons between cultivars/clones and survival year for shoots living in the next year were performed using a Pearson χ2 test. The proportion of two-year surviving short shoots among cultivars and VP clones was compared separately.

	Genotype	Proportion of living short shoots (development years / observation year)		
		2009/2010	2010/2011	2009-10/2011
**Cultivars**	**Bergeron**	0.37 cd	0.36 cd	0.24 bc
	**LE-97**	0.23 cd	0.22 d	0.17 c
	**NS-2**	0.64 ab	0.45 bc	0.36 b
**Velkoplavlovicka clones**	**VP-LE-12/2**	0.46 bc	0.79 a	0.57 a
	**Doc. Blatny**	0.41 cd	0.47 bc	0.38 ab
	**LE-111**	0.44 bc	0.33 cd	0.38 ab
	**LE-130**	0.45 bc	0.32 cd	0.34 b
	**LE-285**	0.29 cd	0.36 cd	0.30 b

The statistical difference among genotypes and particular years was signed with different letters (p = 0.05) with the values.

## Discussion

### Characteristics of the shoot structure

The HSMC estimated for each genotype was composed of three successive states modeling zones with different bud fates from the base to the top of the parent shoot. The relative simplicity in this structure and the low number of zones found is due to the dominance of short and medium shoots in the dataset. Shoots structured in consecutive zones have been previously observed in several Prunus [4, 27, 31] and Malus species ([5, 10]; see [29] for a review).

The basal zone contains latent buds which constitute a pool of buds that enables the tree to react to damages, pruning, and aging by developing epicormics shoots (i.e. shoot that outgrow from dormant buds) [2, 3]. The median zone is composed of central flower buds, which are the result of reproductive differentiation of the buds located at the leaf axils along the parent shoots [23]. The distal zone is composed of vegetative buds as the basal one, but usually those buds allow shoot growth in the next vegetation season. The preferential development of axillary buds close to the top of the parent shoot contributes to acrotony (i.e. long laterals located in the upper part of the parent shoot, for terminology see also [1] and [33]) [32]. The presence of associated flower buds along median and to a lower extent distal zones in adult trees is consistent with previous finding in young apricot, peach and almond trees [27, 28, 29, 31]. This structure was similar for all genotypes and is common for all apricot trees in full bearing stage.

### Differences in shoot structure depending on total shoot length

The analysis of the shoot structure points out differences in the frequency of occurrence and the number of metamers of particular zones as a function of the total number of metamers of the shoot. The systematic occurrence of the basal zone is likely related to preformation [1, 4] building the shoot base [4, 31]. Since the number of preformed organs has been estimated about 10 foliar primordia in peach tree [9], it is likely that a large part of the shoots observed in the present study, located at the third or fourth branching orders was preformed within the winter buds.

The decrease in the number of metamers per shoot mainly from MS to SS or between years led to the shortening or disappearance of the median and/or distal zones. The progressive disappearance of median zone, skipping the development of central flowers and/or the shoot vegetative prolongation has been previously pointed out in apple trees [4, 10]. Despite the lack of correlation between the number of metamers of the shoot and of the median zone, the number of associated flower buds increased with shoot length. The higher number of associated flower buds along the neoformed part of medium and long shoots suggests that these flower buds may differentiate in locations less specific than the central floral buds and during a longer period along the vegetation season. The correlation between the number of metamers of the distal zone and the total number of metamers of the shoot likely results from the timing of growth cessation. The extreme reduction in shoot structure and the disappearance of the distal zone could lead to the mortality of short shoots. Since apricot tree has a tendency to sympodial growth due to frequent apical bud mortality [29, 34], such abortion of apical bud concomitant to the absence of vegetative buds able to regrow in the next spring is likely leading the shoot mortality. Therefore the maintenance of a pool of vegetative buds in the distal zone may be crucial to ensure shoot survival and its growth continuation.

In this study, the year effect was particularly strong, affecting all zones and the total number of metamers of the shoot. The reduction in the total number of metamers per shoot can be partly attributed to tree ontogeny [1, 10]. Moreover, insufficient water or nutrition availability in 2011 (S1 Fig) could have further limited shoot growth, since these two environmental factors influence the whole tree growth [11, 14] and bearing [22, 23, 24].

Altogether, our results suggest that the risk of irregular bearing of the trees could be enhanced when shoot growth is excessively reduced, either by environmental conditions or high crop load [25]. Consistently with previous studies that have shown that the abundance of associated and central flowers is linked to total shoot growth [4, 40], we can assume that 10 metamers per shoot, as mean number across the shoot population, could constitute a minimum number of metamers for maintaining the production in apricot trees, in order to avoid alternate bearing.

### Structural differences among the genotypes

The genotypes differed in their ability to develop the median and distal zone, in the number of metamers of all three zones and in their compositions in terms of central bud fate and number of associated flower buds. One of the main differences among genotypes concerns the median zone. The trees of cvs Bergeron and LE-97 showed in a given conditions higher flowering and bearing potential considering the higher number of central and associated flower buds per shoot. However, the higher production of central flowers within the median zone in SS may negatively affect the distal zone occurrence. Such behavior suggests a different ability of the cultivars (Table 5) to promote central flower buds formation upon the maintenance of vegetative growth in spurs. In particular, LE-97 appears prone to a competition between the bearing potential of the median zone and the distal zone occurrence as it exhibits a long and fruiting median zone (Fig 2, Tables 1 and 4) while the lowest occurrence of the distal zone and lowest rate of SS survival (Fig 2, Tables 5 and 6). This reduced distal zone occurrence, that further promotes the SS mortality rate, can be considered as an additional mechanism controlling the shoot population, like in almond trees [24].

Due to a significant decrease in the number of metamers and frequency of the median zone as well as in the mean number of associated flower buds per shoot in most cultivars and clones, the second year of observation can be considered as an “off” year. The difference among cultivars and clones in the occurrence of the median zone, as well as its fluctuation between the two years is likely to refer, in addition to genetic effect, to several factors such as their physiological reaction to unfavorable environmental conditions in non-irrigated orchard [15, 16] and the fruit load at the branch or tree level [17, 18, 19, 24].

The mentioned differences among the cultivars and clones could further allow differentiating among apricots depending on their flowering abundance and shoot mortality rate, complementing the classification of group 5, as defined by [21]. Since our study was performed on two years only, further analysis of the cultivars at different tree age, in different environmental and orchard management conditions would be relevant.

## Conclusions

In our study, we demonstrated that, in apricot adult trees, shoots are structured in three different and successive zones composed of latent buds (basal zone), flower buds (median zone) and vegetative buds (distal zone). While the basal zone is always present, the last two zones may not develop leading to no flowering or shoot mortality or even both. The median zone length and flower abundance increase with shoot length. The higher number of associated flower buds along the neoformed part of medium and long shoots suggests that these flower buds may differentiate in locations less specific than the central floral buds and during a longer period along the vegetation season. The length of the distal zone likely results from the timing of growth cessation. Our results suggest that the median zone could partly compete with the distal zone, at least in some genotypes, through its flowering/fruiting potential and crop load leading to ealier growth cessation. This mechanism strongly affects short shoot longevity and the maintenance of a minimal shoot length appears crucial to avoid short shoot mortality and alternate bearing.

## Supporting information

S1 Fig Monthly maximum, minimum and mean temperature as well as the monthly amount of rainfall during the shoot development in 2010 and 2011. (TIF)

S2 Fig Three-years-old branch of cv ‘Velkopavlovická’, clone VP-LE-12/2 spring 2011 [37]. (TIF)

S3 Fig Scheme of branching organization and sampling of annual shoots for model estimation. (TIF)

S1 Table Number of shoots and minimum-maximum number of metamers (within the brackets) per shoot type (according to particular length class, i.e. SS, MS and LS), genotype and year. (DOCX)

## Abbreviations

cv/cvs: cultivar/cultivars
HSMC: Hidden semi-Markov chains
LS: long shoot above 10 cm
MS: medium shoots from 2.1–10 cm
SS: short shoot up to 2 cm
VP: Velkopavlovická
