# Supplementary figures and images for: Modelling the bearing and branching behaviors of 1-year-old shoots in apricot genotypes
Source: PLoS One. 2020 Jul 9;15(7):e0235347. doi: 10.1371/journal.pone.0235347 (PMC7347096; doi:10.1371/journal.pone.0235347)

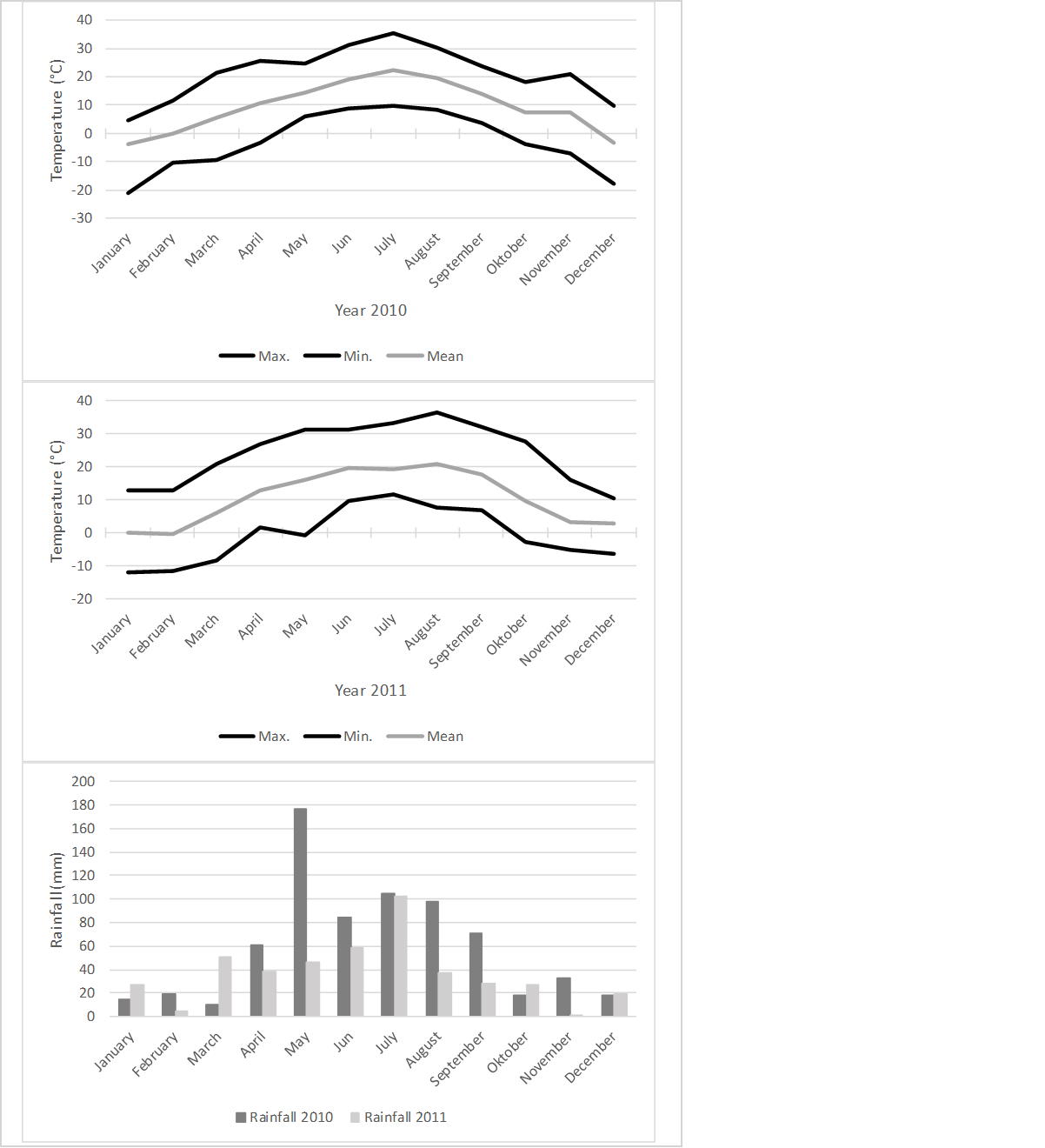

Supplement: S1 Fig — (TIF) [file pone.0235347.s001.tif]

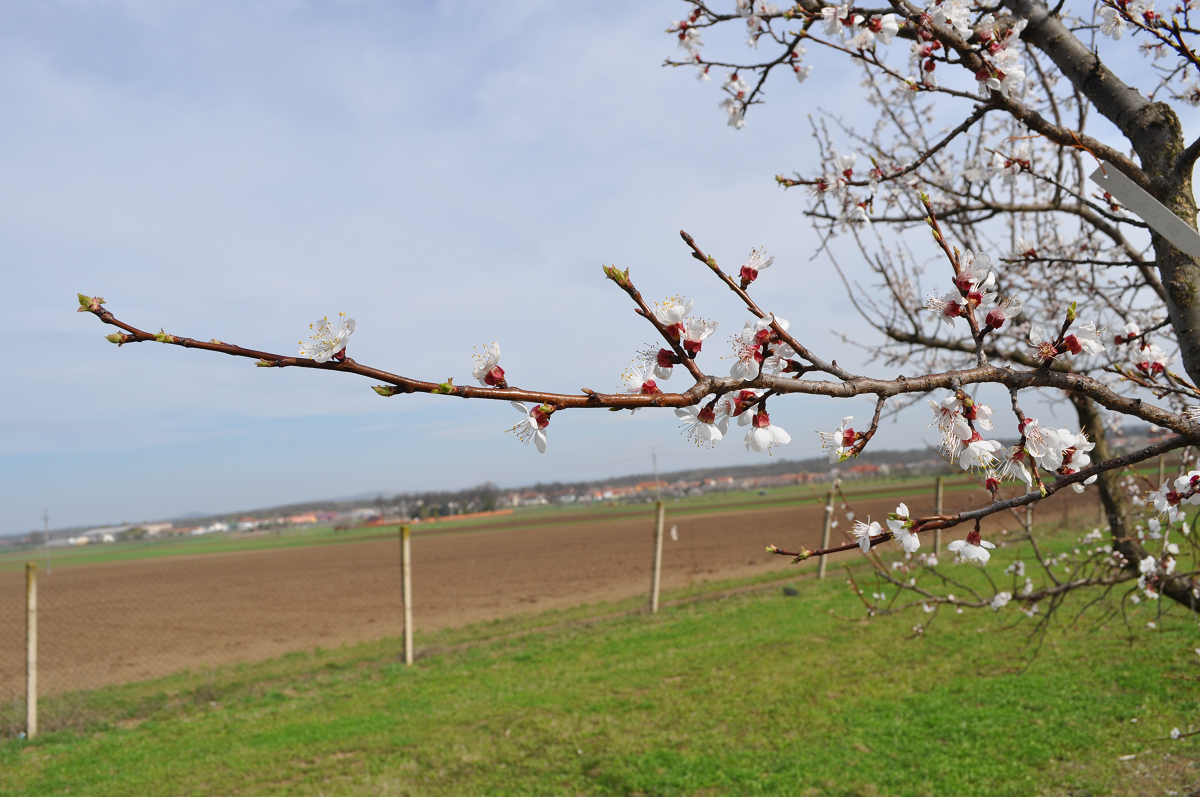

Supplement: S2 Fig — (TIF) [file pone.0235347.s002.tif]

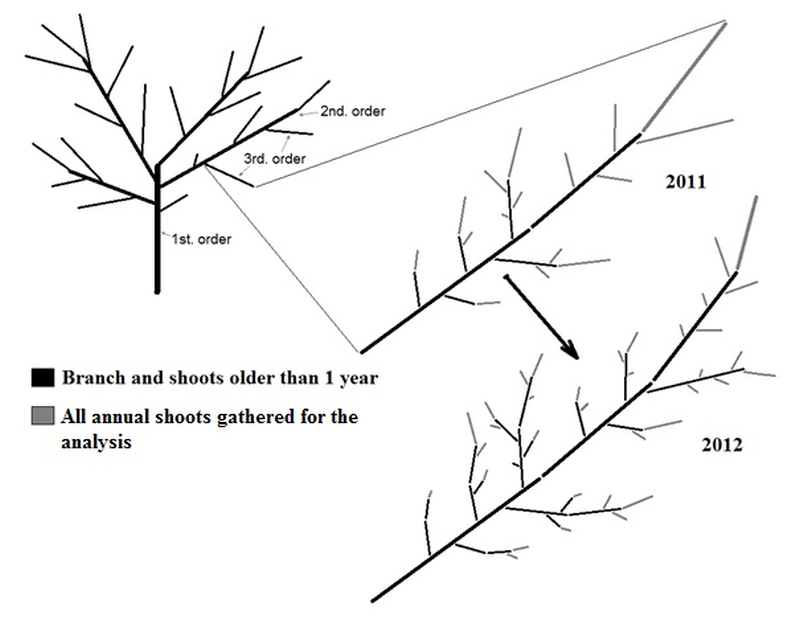

Supplement: S3 Fig — (TIF) [file pone.0235347.s003.tif]
