# Supplementary material for: Modelling the bearing and branching behaviors of 1-year-old shoots in apricot genotypes
Source: PLoS One. 2020 Jul 9;15(7):e0235347. doi: 10.1371/journal.pone.0235347 (PMC7347096; doi:10.1371/journal.pone.0235347)
Supplement: S1 Table — (DOCX) [file pone.0235347.s004.docx]

Table S1. Number of shoots, minimum-maximum number of metamers (into brackets) per shoot type, genotype and year.

|  | **Genotypes** | **SS 2010** | **MS 2010** | **LS 2010** | **SS 2011** | **MS 2011** | **LS 2011** |
| --- | --- | --- | --- | --- | --- | --- | --- |
| **Cultivars** | **Bergeron** | 52 (2-10) | 75 (7-18) | 26 (12-26) | 148 (2-10) | 72 (7-22) | 7 (12-26) |
|  | **LE-97** | 67 (2-10) | 54 (7-16) | 16 (13-24) | 122 (2-8) | 70 (6-15) | 15 (13-20) |
|  | **NS-2** | 57 (2-8) | 61 (6-16) | 15 (12-41) | 119 (2-12) | 60 (8-18) | 14 (14-25) |
| **Velkoplavlovicka clones** | **VP-LE-12/2** | 43 (2-8) | 28 (7-15) | 10 (11-22) | 104 (2-11) | 46 (7-15) | 7 (12-20) |
|  | **Doc. Blatny** | 47 (2-9) | 46 (7-13) | 12 (12-44) | 90 (2-16) | 62 (7-20) | 10 (13-24) |
|  | **LE-111** | 43 (2-9) | 43 (7-15) | 9 (12-20) | 85 (2-12) | 42 (8-14) | 2 (16-18) |
|  | **LE-130** | 59 (2-10) | 73 (7-15) | 11 (12-24) | 129 (2-10) | 74 (7-13) | 7 (11-16) |
|  | **LE-285** | 47 (2-12) | 47 (8-17) | 8 (18-31) | 116 (2-13) | 55 (7-18) | 13 (12-25) |
